# Ecosystem Size Mediates the Effects of Resource Flows on Species Diversity and Ecosystem Function at Different Scales

**DOI:** 10.1002/ece3.70709

**Published:** 2024-12-16

**Authors:** Emanuele Giacomuzzo, Tianna Peller, Isabelle Gounand, Florian Altermatt

**Affiliations:** ^1^ Department of Evolutionary Biology and Environmental Studies University of Zurich Zurich Switzerland; ^2^ Department of Aquatic Ecology Eawag: Swiss Federal Institute of Aquatic Science and Technology Dübendorf Switzerland; ^3^ Institut D'écologie et Des Sciences De L'environnement (iEES Paris) Sorbonne Université, CNRS, UPEC, CNRS, IRD, INRA Paris France

**Keywords:** allochthonous input, detritus, differentiation, disturbance, homogenisation, landscape, nutrients, scaling, species–area relationship, subsidies

## Abstract

Ecosystem size and spatial resource flows are key factors driving species diversity and ecosystem function. However, the question of whether and how these drivers interact has been largely overlooked. Here, we investigated how ecosystem size asymmetry affects species diversity and function of two‐patch meta‐ecosystems connected through flows of nonliving resources. We conducted a microcosm experiment, mimicking resource flows between ecosystems of different sizes yet otherwise identical properties or between ecosystems of the same size. Meta‐ecosystems with asymmetric ecosystem sizes displayed higher α‐diversity but lower β‐diversity and ecosystem function (total biomass) than their unconnected counterparts. At the same time, such an effect was not found for meta‐ecosystems of identical patch sizes. Our work demonstrates how the size of ecosystems, interconnected via resource flows, can modulate cross‐ecosystem dynamics, having implications for species diversity and function across scales.

## Graphical abstract

Ecosystem size and resource flows are key factors driving biodiversity and ecosystem function. However, the question of whether and how these drivers interact has been largely overlooked. Here, we show how ecosystem size asymmetry affects biodiversity and function of two‐patch meta‐ecosystems connected through flows of nonliving resources using a microcosm experiment.

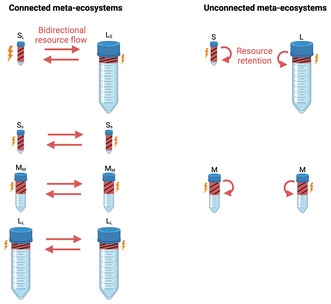

## 1 Introduction

Ecosystem size is a key factor driving species diversity. Ecologists have long known that larger ecosystems harbour more species diversity than smaller ecosystems (species–area relationship; MacArthur and Wilson 1963). The concept dates back to the late 1700s during the second Pacific voyage of James Cook. There, naturalists Johann Reinhold Forster and Georg Forster noted that ‘Islands only produce a greater or less number of species, as their circumference is more or less extensive’ (Forster 1778), which has been empirically and experimentally corroborated many times since (e.g., Fukami 2004; Losos and Ricklefs 2009; Wilson 1961). The various reasons why larger ecosystems harbour more species diversity remains an ongoing area of research (Losos and Ricklefs 2009), and the individual roles of different processes (e.g., speciation and dispersion) contributing to this pattern are still debated (e.g., Valente et al. 2020). The main explanation for this phenomenon has been that species go extinct at lower rates in larger ecosystems (MacArthur and Wilson 1963, 1967) as they have more habitat types (Kallimanis et al. 2008; Williams 1943), more niche diversity (e.g., Ren et al. 2022) and experience less ecological drift (e.g., Gilbert and Levine 2017). The reason why larger ecosystems commonly house more species diversity has been extensively investigated through theoretical, comparative and experimental studies (e.g., Hanski and Ovaskainen 2000; Luo et al. 2022; Wang and Altermatt 2019). Furthermore, ecosystem size can also influence ecosystem function (LeCraw, Romero, and Srivastava 2017; McIntosh et al. 2024; Yang et al. 2021). For example, larger ecosystems can be more productive because communities in larger ecosystems can use resources efficiently thanks to complementary traits (complementarity effects; Delong and Gibert 2019). Additionally, larger ecosystems can support longer food chains (Post 2002), increasing or decreasing ecosystem function according to the trophic level considered (Loreau 2010).

Furthermore, irrespective of their size, ecosystems are rarely isolated in space. Spatial flows and nonliving subsidies among ecosystems (e.g., leaf litter, carcasses, and inorganic nutrients; herein, ‘resource flows’; see Gounand et al. (2018) for a review) are—next to ecosystem size—a key abiotic factor affecting species diversity and ecosystem function. For example, salmon carcasses transported from rivers to land by wolves and bears bring abundant nutrients, which can decrease riparian plant species diversity as they promote the dominance of some species (Hocking and Reynolds 2011). Subsidies from marine algal wrack can either increase plant species diversity on sand dunes (Del Vecchio et al. 2017) or decrease plant species diversity in rainforests on tiny islands (Obrist et al. 2022). As another example, aquatic insects can make up a great part of the diet of riparian birds which feed on them, potentially allowing them to maintain their function (production) (Nakano and Murakami 2001). Supporting such empirical evidence, meta‐ecosystem theory predicts that resource flows can affect species diversity by modifying species interactions and persistence (Gravel et al. 2010; Marleau, Guichard, and Loreau 2014; Peller, Marleau, and Guichard 2022). For example, resource flows can delay competitive exclusion by increasing locally available resources (Gounand et al. 2017) or instead prevent the local establishment of dispersing species by increasing the abundance of the resident competitors (Gravel et al. 2010). Furthermore, resource flows should increase meta‐ecosystem production if they transport resources from ecosystems good at producing biomass through photosynthesis to ecosystems good at transforming nonliving resources into consumers (Harvey et al. 2023). However, despite widespread recognition that ecosystem size and resource flows can affect species diversity and ecosystem function individually, their interactive effect has largely been overlooked.

These two drivers—ecosystem size and resource flows—likely interact since ecosystem size influences the amount and the effect of resource flows. For example, the size of a body of water regulates the amount of resources it exports: The larger a lake or a river, the more insects emerge from it per metre of reach (Gratton and Vander Zanden 2009). Furthermore, the size of the receiving watershed would determine the effects of aquatic resource import: For instance, the larger a watershed, the more diluted its fertilisation from salmon carcasses (Hocking and Reimchen 2009). Also, larger islands that receive algal wrack and carrions from the ocean experience a more diluted positive effect on their secondary production (Polis and Hurd 1995). As resource flows can influence species diversity and ecosystem function, and ecosystem size can influence resource flows, the hypothesis that ecosystem size can influence species diversity and ecosystem function through resource flows emerges naturally as a general concept.

Here, we tested if and how the size of interconnected ecosystems mediates the influence of resource flows on species diversity and ecosystem function using a protist microcosm experiment (Altermatt et al. 2015; Benton et al. 2007; Cadotte and Fukami 2005). We constructed two‐patch meta‐ecosystems connected by resource flows between ecosystems (for clarity we use ‘patch’ as a synonym for ‘ecosystem’). We manipulated (i) the relative size of the two patches within the meta‐ecosystem (symmetric vs. asymmetric sizes) while keeping the total size of the meta‐ecosystem constant, and (ii) the connection between the two ecosystems (connected vs. unconnected). Our results showed as a proof of concept that ecosystem size asymmetry significantly influences species diversity and ecosystem function through resource flows. Specifically, we observed resource flows increasing α‐diversity and a decreasing β‐diversity and ecosystem function (total biomass) in asymmetric meta‐ecosystems when comparing their connected to their unconnected treatment. Contrastingly, resource flows did not affect α‐diversity, β‐diversity or ecosystem function in symmetric meta‐ecosystems, as shown by comparing symmetric connected and unconnected meta‐ecosystems.

## 2 Material and Methods

### 2.1 Experimental Design

**FIGURE 1:**
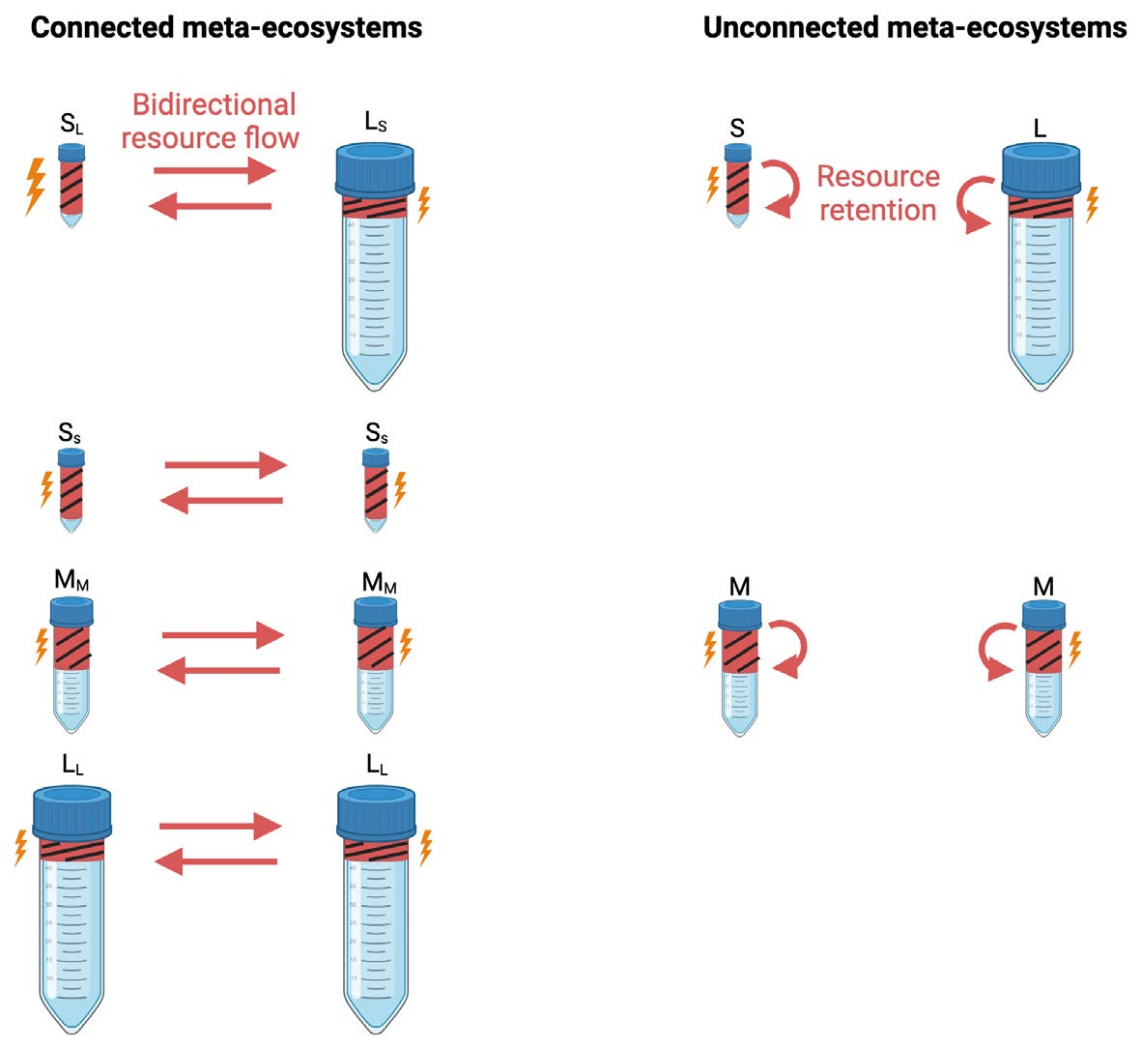
Experimental design. S_L_ = Small ecosystem connected to large ecosystem, L_S_ = large ecosystem connected to small ecosystem, S_S_ = small ecosystem connected to small ecosystem, M_M_ = medium ecosystem connected to medium ecosystem, L_L_ = large ecosystem connected to large ecosystem, S = small unconnected ecosystem, M = medium unconnected ecosystem and L = large unconnected ecosystem. Red portion of the ecosystem with a lightning bolt = Disturbed portion of the community turned into nonliving resources and reciprocally exchanged between ecosystems (in connected ecosystems) or put back into the ecosystem (in unconnected ecosystems). Treatments shown in this figure were crossed with two levels of disturbance, low and high and each treatment combination was replicated five times. This resulted in 110 microcosms paired in 55 meta‐ecosystems.

We studied how asymmetry in ecosystem size mediates the effect of resource flows on species diversity and ecosystem function in meta‐ecosystems via a microcosm experiment involving an aquatic protist community (Altermatt et al. 2015). Specifically, we compared two‐patch meta‐ecosystems with either symmetric or asymmetric sizes (yet identical total meta‐ecosystem size) and either connected by nonliving resource flows or unconnected (see Figure 1). All replicates started with identical initial communities. We evaluated the resource flow effect by comparing connected meta‐ecosystems with pairs of unconnected ecosystems of the same size and symmetry properties (controls, referred to as unconnected meta‐ecosystems). Meta‐ecosystems were of identical total size (volume: 45 mL), with symmetric meta‐ecosystems composed of two identical‐sized patches (each 22.5 mL) and asymmetric meta‐ecosystems composed of a 7.5‐mL and a 37.5‐mL patch, respectively. We call connected symmetric meta‐ecosystems M_M_M_M_ and connected asymmetric meta‐ecosystems S_L_L_s_, with S, M and L referring to small (7.5 mL), medium (22.5 mL) and large (37.5 mL) ecosystems, respectively, and subscripts referring to the size of the connected ecosystem. We call the respective unconnected controls of the resource effect (unconnected meta‐ecosystems) MM and SL, respectively (without subscripts).

To understand the effects of the size of the connected ecosystem, we also established two control meta‐ecosystems connecting two small ecosystems (S_S_S_S_) and two large ecosystems (L_L_L_L_), respectively (see Figure 1), and compared local ecosystems that were connected to either small or large ecosystems. Specifically, we compared S_L_ with S_S_ and L_S_ with L_L_. All the above combinations were replicated fivefold.

### 2.2 Experimental Set‐Up

Our initial communities consisted of eight heterotrophic ciliates (*Blepharisma* sp., *Colpidium* sp., *Loxocephalus* sp., *Paramecium aurelia* , *Paramecium caudatum* , *Spirostomum* sp., *Spirostomum teres,* and *Tetrahymena cf. pyriformis*), two mixotrophic ciliates able to photosynthesise ( *Euglena gracilis* and *Euplotes aediculatus* ), and one rotifer (*Cephalodella* sp.), subsequently all referred to as ‘protists’. We cultured protists in preautoclaved bottles with standard protist medium (0.46 g of protozoa pellet by Carolina per L of water) and a bacterial mix ( *Serratia fonticola* , *Bacillus subtilis,* and *Brevibacillus brevis* ) serving as food for protists and constituting 5% of the total culture volume. See Altermatt et al. (2015) for further details and protocols.

At the start of the experiment (Day 0), we established a master mix of the protist community consisting of all 11 species mixed at 1/11 of their carrying capacity and volumetrically supplemented with 15% standard protist medium. The experiment was conducted in 50‐mL centrifuge tubes, with each tube representing an ecosystem. We pipetted 7.5 mL, 22.5 mL and 37.5 mL of the master mix to constitute the small, medium and large ecosystems, respectively. The replicates were randomised in position and kept in an incubator at 20°C with constant lighting for the remainder of the experiment.

### 2.3 Disturbances and Nonliving Resource Flows

Because it is logistically challenging to separate living species from nonliving resources in aquatic microcosms, spatial flows were associated with disturbances, whose mortality effect was controlled in unconnected ecosystems (Jacquet, Gounand, and Altermatt 2020). Such temporal synchrony of resource flows and disturbances also commonly reflects natural systems in which disturbance and subsequent flows or resources coincide (e.g., forest fire, landslide, etc.). Specifically, every 4 days (starting on Day 5), we boiled a fixed volume of the community of each ecosystem for 30 s to kill every organism, thereby turning all organisms into nonliving resources (i.e., local disturbance). After boiling the sampled volumes in a microwave, we let them cool down to room temperature and then poured them into the connected recipient ecosystems, creating bidirectional resource flows. We investigated whether resource flow level affects our results by considering two levels of disturbances, either boiling 5.25 mL of each ecosystem (low disturbance) or boiling 6.75 mL (high disturbance), which represented 70% and 90% of a small ecosystem, 23.3% and 30% of a medium ecosystem and 14% and 18% of a large ecosystem, respectively, in the low and high disturbance treatments. In the unconnected controls, the same volume was disturbed but poured back to the originating ecosystem to control for the mortality associated with cross‐ecosystem resource flows (‘resource retention’ in Figure 1). We here focus on the results of the high disturbance level. Resource flow effects on species diversity were mediated by patch size at low and high disturbance, but effects on ecosystem function only at high disturbance.

During the experiment, we kept the volume of ecosystems constant by replenishing the inevitable losses of volume that occurred through sampling and evaporation. Three days before each sampling day, we added the protist medium equal to the same volume to be sampled (0.2 mL) to avoid a decrease in total volume. Secondly, we counteracted evaporation losses by replenishing the volumes that evaporated with autoclaved deionised water. Right before the first two disturbances, we added 1.0 mL of deionised water to all tubes right before each disturbance. However, before the third exchange event, we observed slightly higher than anticipated evaporation rates, and the ecosystems were, on average, 1.17 mL (SD = 0.37) smaller than their initial volumes. Therefore, before the third exchange and after each subsequent exchange, we refilled the ecosystems with water until they reached their initial volume.

### 2.4 Sampling

To determine the abundance, species identity, biomass and traits of protists in each ecosystem, we took videos of 0.2 mL samples from each ecosystem every 4 days, starting at Day 0. While the first two time points (Days 0 and 4) took place before the first disturbance, all other time points were always taken 3 days after the disturbance to leave communities to recover as much as possible from disturbances. We took a 5‐s video of each sample at 1.6× magnification, using a Hamamatsu Orca Flash 4.0 (Herrsching am Ammersee, Germany) camera. During the last two time points (Days 24 and 28), we took two samples per ecosystem to reduce the sampling error and increase the chances of detecting individuals at low densities (each metric was averaged across the two samples). We also took videos of all protist monocultures to construct a training dataset of each species' traits for species identification. We took sufficient videos of each monoculture to capture at least 100 individuals of each species.

### 2.5 Quantifying Biomass and Species Diversity

We used the R‐package BEMOVI to identify and characterise protist species in the communities (Altermatt et al. 2015; Pennekamp, Schtickzelle, and Petchey 2015; Pennekamp and Schtickzelle 2013). We first extracted moving particles' traits (e.g., speed, shape and size) in the videos. We then used these traits to filter out particles that were not protists and obtain an average abundance of protist individuals per volume. We also estimated protist live biomass as our focal ecosystem function. We calculated the total area of protists (as area per volume medium) and subsequently used this ‘bioarea’ as a proxy of live biomass (hereafter referred to as ‘biomass’), which is a fair assumption given the roundish shape of protists (see also previous work using the same approximation; e.g., Jacquet, Gounand, and Altermatt 2020; Pennekamp et al. 2018). We then identified protist species using a support vector machine model (Cortes, Vapnik, and Saitta 1995; r‐package ‘e1071’: Dimitriadou et al. 2006), employing traits extracted from species monocultures as predictor variables. Last, we calculated local species diversity (α‐diversity) using the Shannon index. At the meta‐ecosystem scale, we calculated species diversity through (i) mean local species diversity (mean α‐diversity) as the Shannon index averaged across ecosystems, (ii) among‐community species diversity (β‐diversity) as the Bray–Curtis index and (iii) total species diversity (γ‐diversity) as the total number of species persisting at the meta‐ecosystem level.

### 2.6 Statistical Analysis

To understand the effects of resource flows on species diversity over time in symmetric and asymmetric meta‐ecosystems, we performed statistical analysis in R using mixed‐effect models with the ‘lme4’ package (Bates et al. 2015) and post hoc tests with the ‘emmeans’ package (Lenth 2024). The analysis excluded the initial two time points preceding the disturbances (grey zones in all figures), as their inclusion would interfere with our understanding of the impact of disturbances and resource flows.

At the meta‐ecosystem level, to examine resource flow effects, we compared S_L_L_S_ to SL and M_M_M_M_ to MM. SL and MM were virtual meta‐ecosystems created from unconnected ecosystems, that is, pairing two ecosystems to calculate the species diversity and total biomass (without having these ecosystems connected by flows of resources). We constructed these virtual control meta‐ecosystems by creating all possible pairs (without replacement) of unconnected ecosystems (25 SL pairs and 10 MM pairs). To test the influence of the resource flow connection on a response variable (α‐, β‐, γ‐diversity and total biomass), we examined the effects of the resource flow connection and its interaction with time and disturbance level by constructing mixed‐effect models. In the models, random effects considered the impact of the replicates and the baseline (value at the time point before the first disturbance) on the correlated intercept and the slope of the relationship between the response variable and time. We performed multiple comparisons iteratively by bootstrapping 1000 ecosystem combinations, resulting in a distribution of *p* values. Each iteration involved unconnected meta‐ecosystems with differently paired ecosystems (without resampling). We compared meta‐ecosystem types (considering their interaction with time) across disturbance levels performing an ANOVA test followed by a post hoc test with Šidák correction. The presented *p* values are the medians of their respective distributions.

At the local level, we investigated whether the size of the connected ecosystem influenced resource flow effects—comparing S_L_ to S_S_ and L_S_ to L_L_—and whether resource flows had an effect when happening between ecosystems of the same size—comparing S_S_ to S, M_M_ to M and L_L_ to L. To test the influence of the resource flow connection or connected ecosystem size on a response variable (Shannon index and biomass), we made the same model comparisons as at the meta‐ecosystem level (but without iterations).

Also at the local level, to examine the effects of ecosystem size, we investigated whether the size of unconnected ecosystems influenced the ratio between photosynthetic (mixotrophs) and heterotrophic individuals (photosynthetisers–heterotrophs ratio) by comparing S, M and L. Photosynthetic individuals belonged to the species *Euglena gracilis* and *Euplotes aediculatus* . Heterotrophic individuals belonged to the other nine species in the protist community. To test the influence of ecosystem size on this ratio, we examined the effects of ecosystem size and its interaction with disturbance level and time with the same modelling approach and specification we used for the other response variables at a local level.

## 3 Results

**FIGURE 2:**
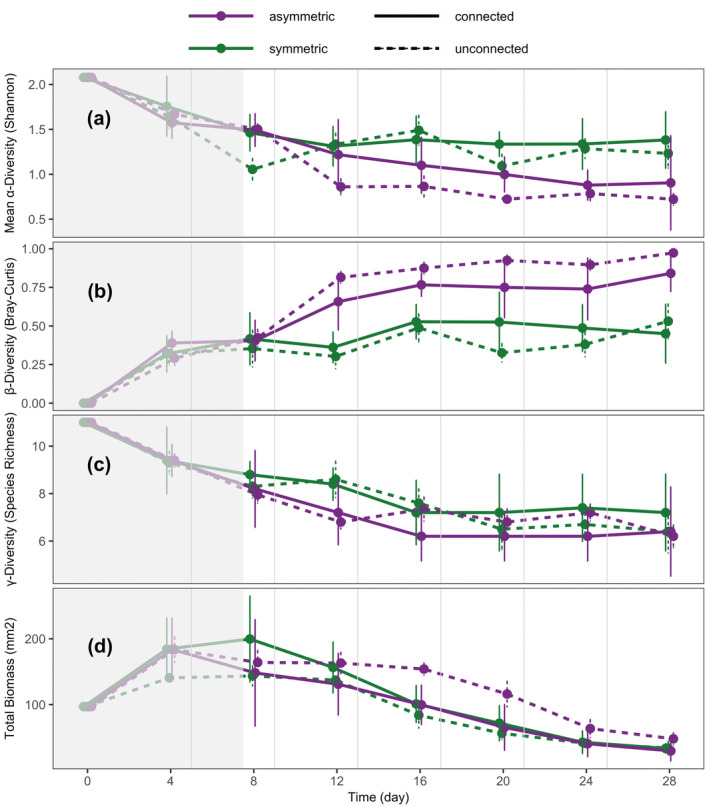
Time series of species diversity and biomass at a meta‐ecosystem scale. (a) Mean α‐diversity (Shannon), (b) β‐diversity (Bray–Curtis), (c) γ‐diversity (species richness) and (d) total biomass (bioarea in mm^2^) of asymmetric and symmetric (purple vs. green) and connected and unconnected (solid vs. dotted lines) meta‐ecosystems. For connected meta‐ecosystems, dots represent means across replicates. For unconnected meta‐ecosystems, dots represent the mean of all possible combinations of unconnected ecosystems assembled as virtual pairs of ecosystems with the respective ecosystem size structure. Error bars represent 95% confidence intervals; vertical grey lines represent disturbance events followed by resource flows. Points were minimally jittered along the *x* axis to make the figure clear. The area in grey indicates time points not considered for analysis as meta‐ecosystems were sampled before the first disturbance and resource flow.

At the meta‐ecosystem level, resource flows increased mean α‐diversity, decreased β‐diversity and decreased total biomass in asymmetric meta‐ecosystems (S_L_L_S_; Figure 2: purple solid vs. dashed lines) but not in symmetric meta‐ecosystems (M_M_M_M_; Figure 2: green solid vs. dashed lines): S_L_L_S_ had a higher mean α‐diversity (*p* = 0.004), lower β‐diversity (*p* = 0.001) and lower total biomass (*p* = 0.004) compared to SL (purple lines in Figure 2a,b,d, respectively). M_M_M_M_ had the same mean α‐ and β‐diversity and total biomass (green lines in Figure 2a,b,d, respectively, *p* > 0.1) as MM. Resource flows did not influence γ‐diversity in either asymmetric or symmetric meta‐ecosystems. That is, S_L_L_S_ had the same γ‐diversity as SL (purple lines in Figure 2c, *p* > 0.1), and M_M_M_M_ had the same γ‐diversity as MM (green lines in Figure 2c, *p* > 0.1).

**FIGURE 3:**
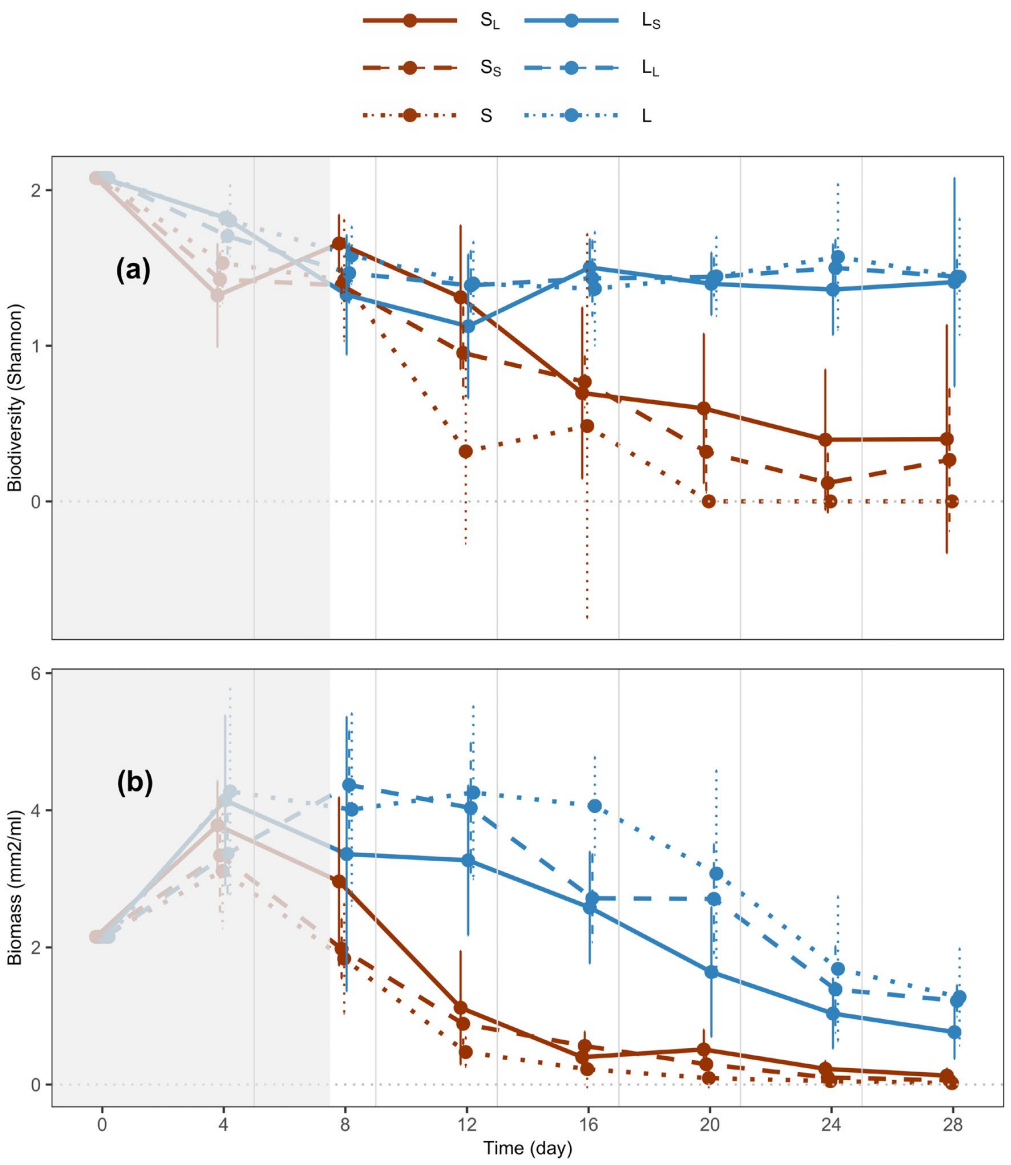
Time series of species diversity and biomass at a local scale. (a) Species diversity (Shannon index) and (b) biomass (bioarea per volume in mm^2^/mL) of small and large ecosystems (red vs. blue lines) connected to an ecosystem of different size, connected to an ecosystem of the same size, or unconnected (solid vs. dashed vs. dotted lines). Medium ecosystems were omitted to make the figure clearer. Dots represent means across replicates; error bars represent 95% confidence intervals; vertical grey lines represent disturbance events followed by resource flows. All systems were sampled on the same day. Points were minimally jittered along the *x* axis to make the figure clear. The area in grey indicates time points not considered for analysis, as ecosystems were sampled before the first disturbance and resource flow.

At the local level, small ecosystems that were connected to large ecosystems (S_L_ vs. S) had higher species diversity (Shannon index) (solid vs. dotted brown lines in Figure 3a, *p* < 0.001) and biomass (but marginally not significant, solid vs. dotted brown lines in Figure 3b, *p* = 0.070) than when unconnected. This effect on species diversity can be broken down into two components. First, the size of the connected ecosystem, as being connected to large ecosystems led to greater species diversity (S_L_ vs. S_S_; solid vs. dashed brown lines in Figure 3a, *p* = 0.001). Second, the presence or absence of the connection as small ecosystems were more diverse when connected to other small ecosystems (dashed vs. dotted brown lines in Figure 3a, *p* = 0.001) than when unconnected (S_S_ vs. S). We did not observe the same trend for biomass. That is, when a small ecosystem was connected to a large ecosystem, it led to the same biomass (solid vs. dashed brown lines in Figure 3b, *p* > 0.1) than when connected to small ecosystems (S_L_ vs. S_S_) and when a small patch was connected to another small patch sustained the same biomass (dashed vs. dotted brown lines in Figure 3b, *p* > 0.1) than when unconnected (S_S_ vs. S).

Also at the local level, large ecosystems that were connected to small ecosystems were similar in their species diversity (solid vs. dotted blue lines in Figure 3a, *p* > 0.1) but had lower biomass (solid vs. dotted blue lines in Figure 3b, *p* < 0.001) relative to when they were unconnected (L_S_ vs. L). For large ecosystems, the connection to small ecosystems decreased their biomass (solid vs. dashed blue lines in Figure 3b, *p* = 0.002) (L_S_ vs. L_L_). This effect was mediated by the size of the connected ecosystem, as when large ecosystems were connected to other large ecosystems, the effect was not observed (dashed vs. dotted blue lines in Figure 3b, *p* > 0.1) (L_L_ vs. L).

**FIGURE 4:**
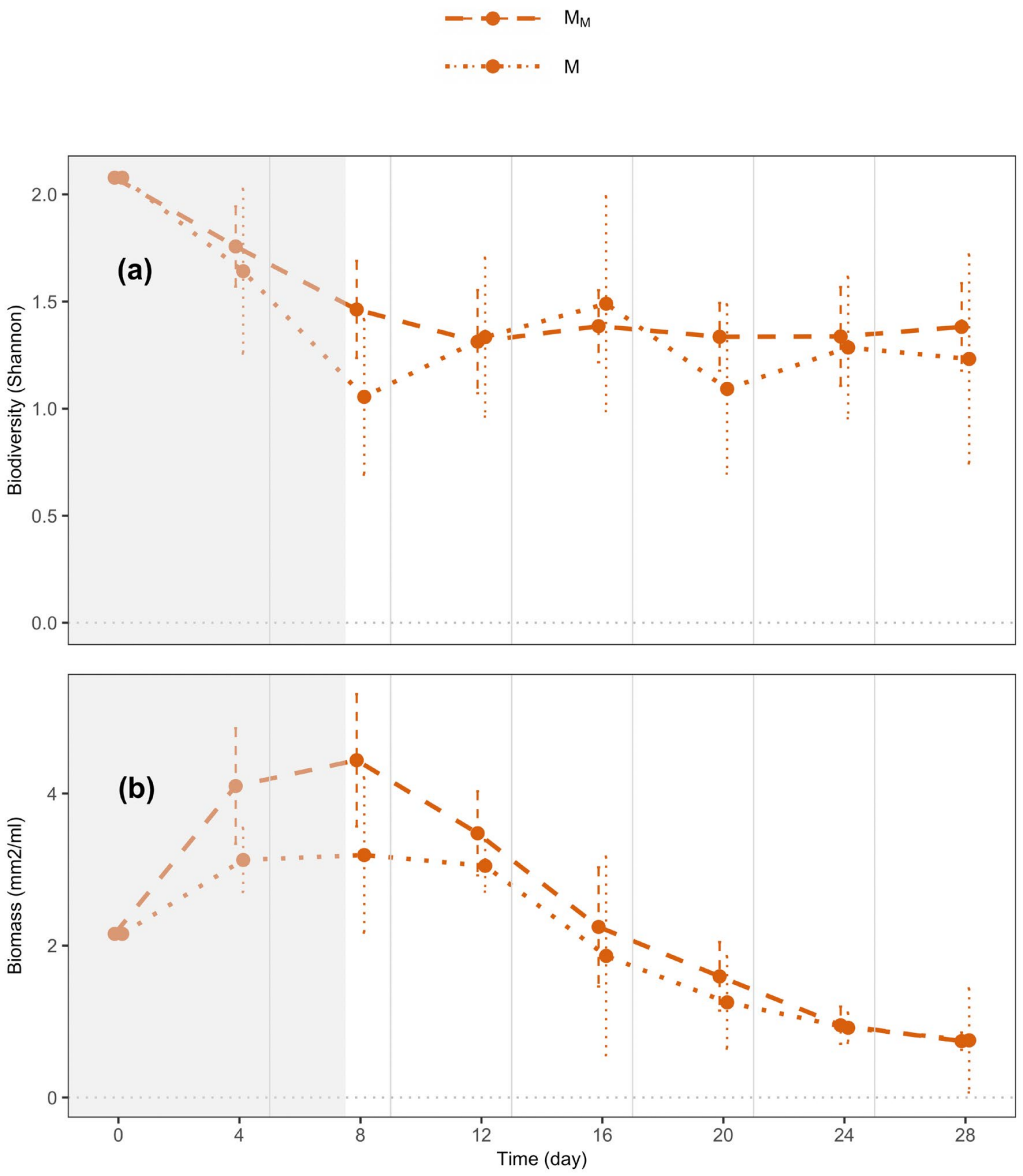
Time series of species diversity and biomass at a local scale. (a) Species diversity (Shannon index) and (b) biomass (bioarea per volume in mm^2^/mL) of medium ecosystems connected to another medium ecosystem or unconnected (dashed vs. dotted lines). Dots represent means across replicates; error bars represent 95% confidence intervals; and vertical grey lines represent disturbance events followed by resource flows. All systems were sampled on the same day. Points were minimally jittered along the *x* axis to make the figure clear. The area in grey indicates time points not considered for analysis, as meta‐ecosystems were sampled before the first disturbance and resource flow.

At the local level as well, in medium ecosystems, we observed a weak, yet marginally nonsignificant, trend of resource flows increasing species diversity (Figure 4, *p* = 0.070) and a significant trend increasing biomass (Figure 4, *p* = 0.043) in connected ecosystems relative to those that were unconnected (M_M_ vs. M).

**FIGURE 5:**
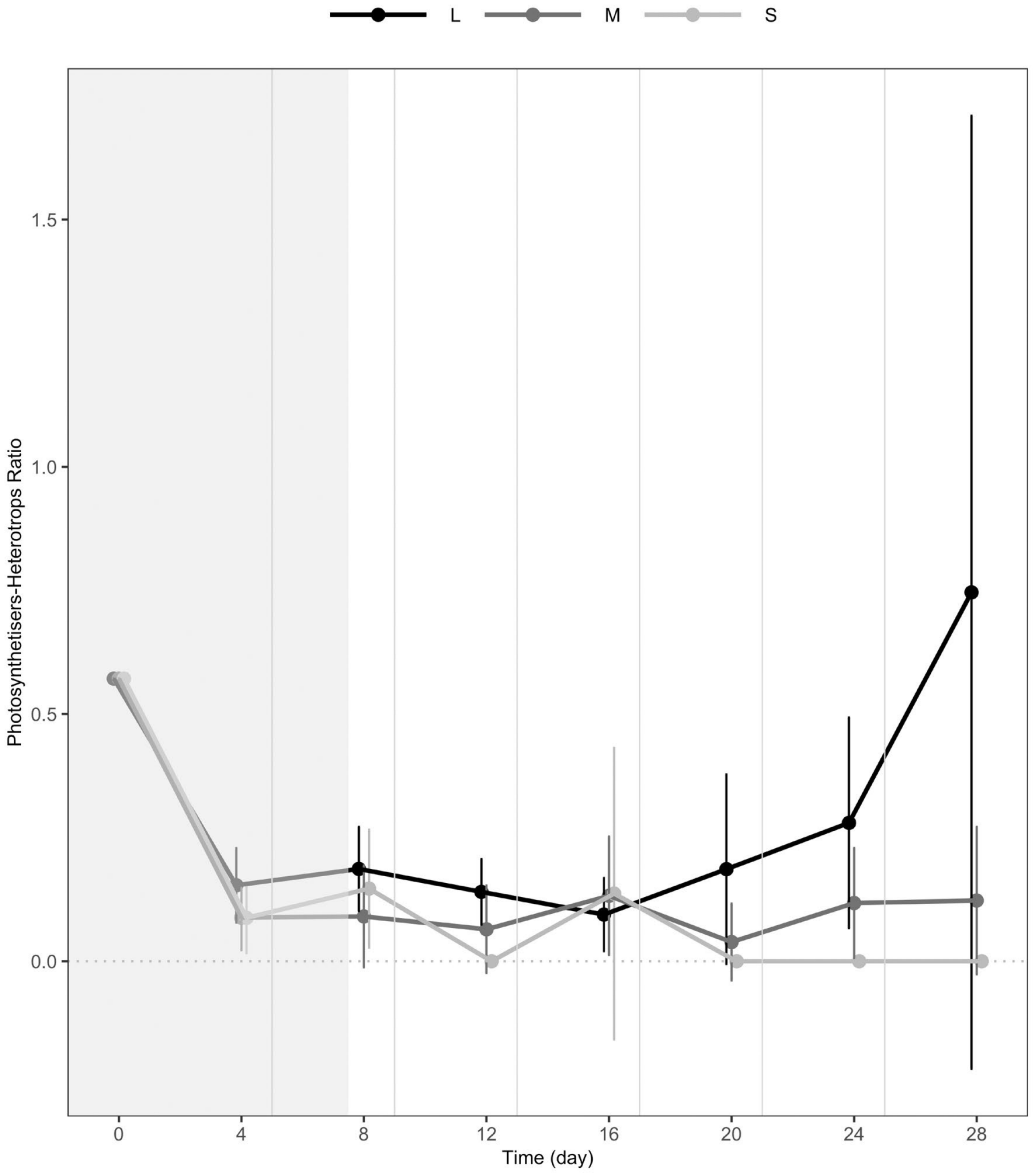
Time series of the photosynthetisers–heterotrophs ratio at a local scale in small, medium and large (light grey vs. dark grey vs. black lines) unconnected ecosystems. Photosynthetisers refer to mixotrophic individuals able to photosynthesise. Dots represent means across replicates; error bars represent 95% confidence intervals; and vertical grey lines represent disturbance events followed by resource flows. All systems were sampled on the same day. Points were minimally jittered along the *x*‐axis to make the figure clear. The area in grey indicates time points not considered for analysis as meta‐ecosystems were sampled before the first disturbance and resource flow.

Finally, larger ecosystems were denser with photosynthetic individuals, as attested by the photosynthetisers–heterotrophs ratio increasing with ecosystem size from small to large unconnected ecosystems (light grey vs. black lines in Figure 5, *p* < 0.001) (S vs. L). This ratio did not increase from small to medium unconnected ecosystems (light grey vs. dark grey lines in Figure 5, *p* > 0.1) (S vs. M).

## 4 Discussion

We experimentally demonstrate in a proof‐of‐concept study that ecosystem size asymmetry can mediate the effects of bidirectional resource flows on species diversity and ecosystem function. Meta‐ecosystems with asymmetric patch sizes (S_L_L_S_) had more similar communities (lower β‐diversity) and lower function (lower total biomass) but maintained higher species diversity across the two local patches (higher mean α‐diversity) than asymmetric yet unconnected meta‐ecosystems (SL). These effects were not observed in meta‐ecosystems with symmetric ecosystem sizes, indicating a mediating role of ecosystem patch size. These results could be explained by the connection of a small ecosystem to a larger ecosystem resulting in an increase in species diversity in the small ecosystem (S_L_ had greater species diversity than S_S_ and S) while leaving the species diversity of the large ecosystem unchanged (species diversity was similar between L_s_, L_L_ and L) and decreasing the biomass of the large ecosystem (L_S_ had lower biomass than L_L_ and L). As small and large ecosystems at the beginning of the experiment were identical aside from their size (resource concentration, community composition, etc.), the effects of the connection can be attributed to ecosystem size. Ultimately, our findings suggest that considering the size of interconnected ecosystems can help us understand how bidirectional resource flows shape species diversity and ecosystem function.

Notably, we found that resources flowing between ecosystems of different sizes impacted both α‐ and β‐diversity by increasing the species diversity of the smaller patch within the meta‐ecosystem. Furthermore, they decreased total meta‐ecosystem biomass by decreasing the biomass of large patches, which was not compensated by the congruent increase in biomass in small patches. We suggest three mutually nonexclusive mechanisms by which small patches may have gained species diversity and biomass while large patches lost biomass compared to the unconnected control patches. All mechanisms should be the result of the effects of a change in resources coming from a difference between the resources gained by the inflow of resources and resources lost by the outflow of resources, as we precluded dispersal.

First, resource quantity: Small ecosystems may have had a net import of resources. Although the volume exchanged between ecosystems was identical, larger ecosystems had a greater dominance of photosynthetic species than small ecosystems (Figure 5), which might have increased carbon availability more in large versus small ecosystems. Consequently, small ecosystems may have imported a greater quantity of sequestered carbon from large ecosystems relative to what they exported, creating an emergent source–sink dynamics of resources (sensu Gravel et al 2010 and Loreau et al. 2013). This net import of resources could have allowed small ecosystems to sustain more species diversity as more resources allow more individuals to persist, promoting a greater abundance of rare species and preventing their extinction (species energy theory, see Wright 1983). The net import of C‐rich necromass to small patches and net export from large patches could have increased basal resources for bacteria—primary resources of protists—in small patches at the detriment of large patches, which cascaded up on biomass production: The decrease in meta‐ecosystem biomass caused by a larger decrease in biomass in large patches than the increase in biomass in small patches could be explained by a net movement of resources to the small patch, which could have had lower recycling rates. Indeed, higher recycling rates in larger patches are reasonable to expect, as they can be found in nature (Donghao et al. 2021; LeCraw, Romero, and Srivastava 2017; Yang et al. 2021). In natural ecosystems, we would expect differences in ecosystem sizes to lead to differences in the quantity of resources exchanged as well, potentially through different mechanisms. For instance, the trophic island biogeography theory (Gravel et al. 2011; Holt 2009) predicts variation in the ratio between autotrophs and consumers between ecosystems of different sizes. Gravel et al. (2011) supported this prediction by parameterising a trophic metacommunity model using 50 pelagic food webs (Havens 1992) and showing that larger ecosystems contained more consumers relative to autotrophs. The explanation for this result is that consumers are more likely to find one of their prey in larger ecosystems and, therefore, establish.

Second, resource quality: Small ecosystems may have had a net import of detritus (protist detritus) of better/different quality. If the detritus of protists was of higher quality as a resource for the local community compared to other resource forms (e.g., bacterial detritus and inorganic nutrients), it would have sustained a higher growth of individuals and, therefore, higher species diversity in the small ecosystem. Consequently, the movement of resources of higher quality to the small ecosystem and of lower quality to the large ecosystem would have increased the function of small ecosystems and decreased the function of the large ecosystem, as a meta‐ecosystem model showed that good quality subsidies should increase the function of the receiving ecosystem and bad‐quality subsidies should decrease it (Osakpolor et al. 2023). We would also expect this mechanism, where size differences between connected ecosystems create differences in the quality of resources exchanged and cascade to influence species diversity and function, to occur in natural ecosystems, potentially through different mechanisms. For example, ecosystems of different sizes can have different biomass distributions across trophic levels (Petermann et al. 2015), with often higher maximal trophic levels in larger ecosystems (Guo et al. 2023; Post, Pace, and Hairston 2000; Ward and McCann 2017). Moreover, different trophic levels might produce detritus of different qualities as consumers often have higher nitrogen content than producers (Elser et al. 2000). As a consequence, the relative quantities of biomass in trophic levels determine the overall quality of the resources exchanged with other ecosystems, which depends on ecosystem size.

Third, resource heterogeneity: Small ecosystems might have imported resources that were more heterogeneous than their own. As there was greater protist species diversity in large than in small ecosystems, the corresponding exported detritus might have been more diverse with respect to carbon compounds and biomolecules, potentially creating more niches for protists to coexist in small ecosystems (resource diversity hypothesis, Lawton 1983). The positive correlation between detritus heterogeneity and consumer feeding on it has been observed in nature (Moore and William Hunt 1988; Yodzis 1988). We expect that also in nature differences in ecosystem size would cause differences in resource heterogeneity and, therefore, cause resource flows to influence species diversity and ecosystem function. Larger ecosystems generally have higher species diversity within trophic levels (horizontal diversity, MacArthur and Wilson 1963, 1967) and higher number of trophic levels (vertical diversity or maximum food chain length, Guo et al. 2023; McHugh et al. 2015; Post, Pace, and Hairston 2000; Ward and McCann 2017). Such higher species diversity should translate into a change in biomass composition (e.g., species diversity can be related to stoichiometry; Striebel, Behl, and Stibor 2009) and higher resource heterogeneity, which would constitute more heterogeneous resources that would determine the effects of resource flow on species diversity and function. Our study highlights that the size of the donor ecosystem, where resource flows originate, can shape the effect of resource flows on a recipient ecosystem's species diversity. In particular, in our experiment, species diversity increased in ecosystems with small patch sizes when connected to ecosystems of large patch sizes more than when connected to ecosystems of small patch sizes.

The subsidised island biogeography theory (Anderson and Wait 2001) states that resources flowing into an ecosystem can influence its species diversity, making its species diversity deviate from what we would expect from species–area relationships, especially in small ecosystems. There is some comparative evidence by field studies, for instance, with resource flows increasing the species diversity of bird species more on smaller than on larger islands (Obrist et al. 2020). However, experimental evidence of this phenomenon is largely lacking. Here, we give a formal experimental corroboration that resources exchanged between differently sized ecosystems affect species diversity and ecosystem functions (e.g., biomass) and are modulated by the differential patch size. In particular, we highlight and discuss how the size of the exporter ecosystem may mediate the quantity, quality and heterogeneity of resource flows through various mechanisms that would modulate the effect on the diversity and functioning of the recipient ecosystem.

Decades of research on spatial subsidies have documented that donor ecosystems commonly vary in size. For example, islands which export nitrogen to coral reefs (Lorrain et al. 2017), kelp forests which exchange nonliving resources with their adjacent intertidal zone (Tallis 2009) or forests that export leaf litter to streams (Larsen, Muehlbauer, and Marti 2016). Moreover, evidence from natural systems supports our finding that donor ecosystems' size can influence recipient ecosystems' species diversity and function. Such evidence is found in lakes and rivers embedded in terrestrial watersheds of different sizes. Notably, studies found that larger watersheds can (i) increase lake primary production, as they export more phosphorus (Knoll, Vanni, and Renwick 2003), (ii) sustain fewer lake consumers that rely on sediments, as they export lower quantities of sediments (lower water flow, gentler slopes and increased sedimentation in terrestrial ecosystems) (Babler, Pilati, and Vanni 2011), and (iii) sustain longer river food chains, as they have more water flow, hence less hydrological variation and therefore a more stable environment (Sabo et al. 2010). This, in conjunction with our findings, suggests that subsidised island biogeography (Anderson and Wait 2001) would gain in integrating how the size of the connected ecosystems mediates the effects of resources on the shape of species–area relationships and possibly changes this relationship. According to our results, we expect, for example, that the species diversity of macroinvertebrates in a lake might be higher than expected by their area only (according to subsidised island biogeography) when the lake is connected to a larger rather than a small forest.

In conclusion, our experiment provides experimental proof of concept that asymmetry in ecosystem size can indirectly affect species diversity and function in meta‐ecosystems through its effects on a ubiquitous connection among ecosystems–spatial flows of resources. Consequently, this implies a need to consider how ecosystem size changes resource flow between ecosystems when aiming to generally understand the drivers of species diversity and ecosystem function in spatially structured systems. Future research should focus on how ecosystem size impacts meta‐ecosystems through resource flows, testing our proposed mechanisms on resource quality and heterogeneity in relation to species diversity and incorporating other properties of resource flows, such as asynchronous flows (Nakano and Murakami 2001), as well as the magnitude of resource flow in relation to ecosystem size (e.g., Gratton and Vander Zanden 2009).

## Author Contributions

**Emanuele Giacomuzzo:** conceptualization (equal), data curation (lead), formal analysis (lead), methodology (equal), project administration (lead), software (lead), visualization (lead), writing – original draft (lead), writing – review and editing (lead). **Tianna Peller:** conceptualization (equal), supervision (equal), writing – original draft (equal), writing – review and editing (equal). **Isabelle Gounand:** conceptualization (equal), funding acquisition (equal), supervision (equal), writing – original draft (equal), writing – review and editing (equal). **Florian Altermatt:** conceptualization (equal), funding acquisition (lead), project administration (lead), resources (lead), supervision (lead), writing – original draft (equal), writing – review and editing (equal).

## Conflicts of Interest

The authors declare no conflicts of interest.

## Data Availability

The data that support the findings of this study are openly available in Zenodo at https://doi.org/10.5281/zenodo.14054339.
